# Factors involving in healthcare professionals’ decision-making process regarding the use of restrictive care practices in adult mental health inpatient units: A protocol for an umbrella review

**DOI:** 10.1371/journal.pone.0319228

**Published:** 2025-02-24

**Authors:** Zelalem Belayneh, Biazin Yenealem, Alemayehu Molla, Nigus Alemnew, Temesgien Ergetie, Workua Mekonnen, Birhanie Mekuriaw

**Affiliations:** 1 Department of Psychiatry, Dilla University, College of Health and Medical Sciences, Dilla, Ethiopia; 2 School of Primary and Allied Health Care, Monash University, Melbourne, Australia; 3 Department of Psychiatry, Injibara University, College of Health and Medical Sciences, Injibara, Ethiopia; 4 Minilik II Medical and Health Science College, Addis Ababa, Ethiopia; 5 Department of Psychiatry, Bahir Dar University, College of Medicine and Health Sciences, Bahir Dar, Ethiopia; 6 Amanuel Mental Specialized Hospital, Addis Ababa, Ethiopia; 7 Faculty of Health, University of Technology Sydney, Sydney, Australia; NYU Grossman School of Medicine: New York University School of Medicine, UNITED STATES OF AMERICA

## Abstract

**Introduction:**

The use of restrictive care practices, such as physical restraint, seclusion, and chemical restraint, remains common practices, yet controversial aspect of mental health care, particularly in inpatient settings. Variations in legal definitions and protocols guiding the implementation of these practices often create legal and ethical dilemmas for professionals when deciding whether to implement such practices in certain circumstances. This leads them to rely on subjective decision-making, which is further shaped by epistemic disparities stemming from differences in knowledge, perspectives, and experiences that impact professionals’ understanding and implementation of restrictive practices.

**Aims:**

This is a study protocol for an upcoming umbrella review aiming to identify and analyse the thematic elements that affect healthcare professionals’ decision-making regarding the uimplementations of restrictive care practices in adult mental health inpatient settings.

**Methods:**

This protocol outlines the approach for an umbrella review. The review will conduct a systematic literature search of electronic databases (Scopus, CINAHL, Embase, OVID, PsycINFO, Cochrane Reviews, and Web of Science) using comprehensive search strings, as well as a manual review of reference lists from included reviews. Only review studies will be considered. Textual data discussing issues related to professionals’ decision-making regarding the use of restrictive care practices will be extracted from each study, coded, and analysed using inductive content analysis. This protocol has been reported according to the PRISMA-P Checklist.

**Discussion:**

The findings from this review will provide valuable insights into the key factors influencing the implementation of restrictive practices in adult mental health inpatient settings. The factors identified in this umbrella review will be applied in clinical practices to guide healthcare practitioners in considering and navigating various contexts when deciding how and in which situations restrictive care practices should be implemented. This contributes to the current global effort in promoting more patient-centered, transparent, evidence-based and accountable practices, while minimizing restrictive interventions. It will also inform future training, practice, and policy interventions aiming at reducing the use of restrictive measures in adult mental health settings.

**Protocol registration:** This protocol has been registered in PROSPERO (CRD-42024581848).

## Introduction

In mental health settings, practices such as seclusion, physical restraint, and chemical restraint are often considered necessary to manage acute behavioral disturbances and ensure the safety of both patients and staff [1,2]. These practices, collectively referred to as restrictive care practices, limit an individual’s freedom of movement, choice, and independence [3]. However, their use is highly controversial, raising significant ethical and clinical concerns due to the immediate and long-term physical and psychological harm they can cause[4].

The use of restrictive care practices can lead to increased psychological distress, worsening of mental health symptoms, re-traumatization, erosion of trust and therapeutic relationships, and various physical complications, including injuries, falls, and even death [5]

One study indicated that between 25% to 47% of individuals subjected to seclusion or restraint could develop post-traumatic stress disorder as a result of these interventions [4]. The negative impact of using restrictive care practices is not limited to service users; health professionals and family members may also experience distress from being involved in or witnessing the implementation of these practices in mental health settings [6].

Over the past 20 years, there is a widespread belief that restrictive practices are non-therapeutic and should be minimized [7]. These practices are recommended to be used only for very brief periods when all other less restrictive options have been attempted and proven ineffective in a given situation [8]. To decrease the reliance on these practices and advance the implementation of lesser restrictive care approaches, various intervention strategies and policy reforms have been implemented worldwide [9,10]. However, the variability in the reported rates of these practices makes it difficult to make valid comparisons of data to evaluate the effectiveness of these strategies and policy reforms for reducing the use of restrictive care practices at the international level [11,12]. Some regions reported evidence of reducing the use of restrictive care practices, while others reported rising rates [13,14]. For instance, a recent systematic review and meta-analysis found that the prevalence of physical restraint ranged from 0.35% to 56%, seclusion from 2.1% to 55.8%, and chemical restraint from 1.9% to 63% [12]. When clustering the physical restraint data across different regions, studies from North America (8%), Australia (9%), and Europe (12%) had lower prevalence rate compared to those from Asian (29%) and African (32%) countries.This makes it difficult to draw meaningful conclusions and understand the true nature of these practices [15]. These variations may stem from differences in how restrictive care practices are implemented across different regions and between professionals [11,16].

**Fig 1:**
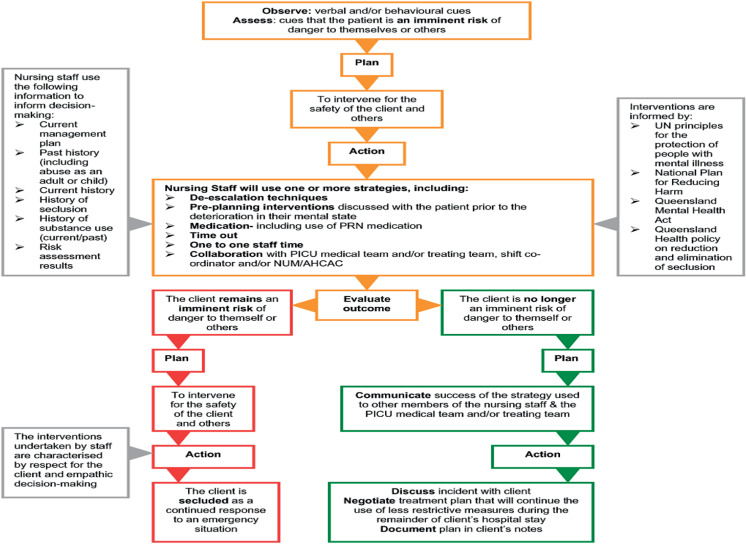
Conceptual framework for professional's decision-making in the use of restrictive care practices in mental health settings.

Healthcare professional's clinical decision-making is an essential component of mental health nursing practice [17]. However, the decision-making process in the use of restrictive care practices, particularly in mental health settings is both complex and dynamic, often surounded by multilyered factors leading professionals to ethical dilemmas [18,19]. Fig 1 below illustrates how professionals balance ethics, safety, and legal requirements during the implementation of restrictive care practices in mental health facilities. This framework emphasizes integrating diverse service users’ perspectives and preferences regarding restrictive care practices with the professional clinical judgment during the decision-making process.

The flowchart in Fig 1 is originally developed by Hyde, Fulbrook, et al. (2009) in Queensland, Australia [20]. This model highlights how professionals navigate decision-making in mental health inpatient settings. It demonstrates the involvement of multiple factors in the clinical decision-making process, aiming to balance professional judgment with ethical and legal requirements, as well as service users’ preferences [20].

The significant variations in the policies and protocols guiding the implementation of restrictive care practices across different regions pose significant challenges [21]. For example, the use of rapid tranquilizers is considered a routine part of care in some regions, such as in developing countries like Ethiopia. In contrast, this practice is regarded as restrictive and more corvive that requires documentation in hospital incident reporting systems in other countries. Such variations also exist between different regions and settings within the same country. For instance, the policies and guidelines regarding the legal definition and reporting mechanisms for chemical restraint vary across the different states of Australia. Moreover, the existence of different definitions of what constitutes restrictive care practices leads professionals to apply their own subjective interpretations to determine when and in what contexts restrictive care practices are necessary, resulting in inconsistencies in clinical practice [22]. A recent qualitative review highlighted the lack of standardized definitions for various forms of restrictive care practices and revealed significant variations in the terminology and conceptual boundaries used to define the constructs of physical restraint, seclusion, and chemical restraint in adult mental health inpatient settings [22]. This creates a situation wherethe same condition might be managed differently across different hospitals or even different healthcare professions within the same hospital [23]. TSuchinconsistency hinders efforts to promote less restrictive interventions, and professionals could inadvertently replace one type of restricitve care practicewith another, potentially more coercive methods, while intending they are implementing a less restrictive alternative [24]. There is no common agreement on what is less restrictive and the determination of which intervention is less restrictive often depends on the professional’s subjective viewpoint [2].

The decision-making process for implementing restrictive care practices in clinical settings is shaped by several interrelated factors [25]. While several previous systematic reviews have identified numerous contexts influencing professionals’ decision-making, their findings are often limited to specific professionals, restrictive geographicalsettings or focused on only certain forms of restrictive care practices. A more comprehensive understanding of the overall situations that could impact decision-making is needed, involving different professional settings and various techniques of restrictive care practices, considering the international contexts [26]. This broader perspective would offer more precise and comprehensive insights into the potential markers leading to discrepancies in clinical practice when apllying restrictive care practices. Understanding these factors is crucial for identifying future training, practice, and policy needs, as well as for designing practical intervention programs to effectively minimize the us eof restricitve care practices [27].

### Aims

This is a study protocol for a subsequent umbrella review aiming to systematically examine existing litrature reviews and synthesize evidence on the conditions and facors that influence professionals’ decision-making processes in the use of restrcitve care pracvtices. By consolidating evidence on the factors that shape these decisions, this umbrella review can promote consistency in clinical practice and pinpoint areas in need of training or policy intervention [28]. Conducting an umbrella review is more efficient than undertaking multiple individual reviews, as it consolidates existing research into a single, accessible document [29].

## Methods

### Study design

This study protocol is a plan for a future umbrella review and does not generate or report any findings; instead, the whole content of this manuscript consists of plans for an upcoming umbrella review that has not been started yet [30]. The umbrella review method has been selected to facilitate intuitive conclusions across variables [31], enabling a comprehensive analysis and integration of existing evidence by synthesizing previously published systematic reviews [32]. The protocol for this review has been registered in the International Prospective Register of Systematic Reviews (PROSPERO) [33]: CRD42024581848. This protocol is now reported according to the PRISMA-P Explanation Checklist (S1 File).

### Data sources and searching methods

We plan to conduct a systematic search of the literature across seven health science electronic databases: MEDLINE, Scopus, CINAHL, Embase, PsycINFO, Web of Science Collections, and the Cochrane Database of Systematic Reviews. The search terms and strategies will be guided by the Population, Intervention, Context, and Outcome (PICO) framework [34]. “Population” will include terms related to healthcare professionals working at inpatient mental health settings, “Intervention” will focus on various forms of restrictive care practice techiniques, “Contexts” will represent mental health inpatient settings, and “Outcome” will address concepts related to factors involving in healthcare professionals decision-making to use these practices (**Table 1**).

**Table 1:** Concepts and key-words that will be used for the search strings of the upcoming umbrella review.

The Population, Intervention, Context and Outcome-PICO approach			
**P-**Population	**I-**Intervention	**C-**Context	**O-**Outcome
“Nurs*” “Professional*” “Staff” “Clinici*” “Psych*” “Health care*” “Doctor*” “Social work*”	“Restrictive practice*” “Restrictive care” “Holding*” “Immobilization*” “Restrictive intervention*” “Restraint*” “Seclusion*” “Confinement” “Coerc*” “Pausing” “Sedation” “Tranquil*” “Forced medication” “Involuntary admission*” “Involuntary treatment*”	“Mental health” “Mental illness*” “Mental disorder*” “Mental disease” “Psych*” “Schizophrenia” “Bi?polar disorder*” “Depression” “Anxiety disorder*” “Posttraumatic stress disorder” “Mentally ill person*” “People with mental illness” “Mood disorder*” “Mania*”	“Decision*” “Preference*” “Practice*” “Experience*” “Attitude*” “Perception*” “Use” “Determin*” “Select*”

A comprehensive search string will be developed using key-words and MeSH terms related to each review concept. Additionally, Boolean operators (e.g., AND, OR, NOT) and truncation symbols (e.g., #, $, *, “) will be employed according to the specific requirements of each database to construct complex search strings that capture variations in word endings and spellings [35]. The search process will include studies conducted without geographical restrictions to captre any review from any part of the world. However, only review studies published in English or those with English-language translation will be considered. This restriction has been implemented due to the lack of bi-lingual interpreters within the research team and because of budget limitations and no access to translators. The research team acknowledges the potential for language bias and addresses this limitation in the discussion section of the paper. The review also covers a broad range of restrictive care practices, inlcluding physicalr esraint, chemical restraint, mechnical restraint, secusion, involuntary admission, foreced medication, emotional and psychological restraint and other forms of coercive measure. This will provide insights into the diverse contexts aloowing for clear understanding of factors involving in healthcare professionals’ decision- making when aplying these practices. We will also manually check the reference lists of included studies to identify potentially eligible reviews to be included in this umbrella review. The final search strategy will undergo a pilot test to assess its effectiveness in identifying relevant studies, with appropriate modifications made if necessary.

### Plan for inclusion criteria and study selection

The eligibility of each study will be assessed using the Sample, Phenomenon of Interest, Design, Evaluation, and Research type (SPIDER) framework [36] (**Table 2**). The SPIDER tool provides an alternative to the commonly used PICO (Population, Intervention, Comparison, Outcome) framework by modifying its components, making it more appropriate for identifying qualitative research studies [39]. This review will also exclude studies conducted among children and older adults due to the contextual variations and differences in legal attributes that guide the use of restrictive care practices in these population groups compared to adult inpatient facilities. Therefore, the findings of this review will specifically apply to adult mental health inpatient settings.

**Table 2:** SPIDER eligibility assessment criteria.

SPIDER framework items	Descriptions
S-Sample Participants	Mental health professionals working in adult inpatient settings
PI-Phenomenon of Interest	Use of restrictive care practices
D-Design	Systematic reviews or meta-analyses of qualitative, quantitative, or mixed-methods studies having qualitative data
E-Evaluation	Factors and contexts influencing decision-making regarding the use of restrictive practices
R-Research Type	Umbrella review

Note: **RCPs** in this review are broadly defined to include restraints (mechanical, physical, or chemical), seclusion, forced medication, involuntary admission and other coercive measures.

Professionals: Refers to any type of healthcare professional working in adult, mental health, inpatient settings.

**Exclusion**: Studies conducted in outpatient or non-adult mental health inpatient settings, such as paediatric, geriatric, and forensic units will be excluded due to the distinct circumstances and legal considerations surrounding the implementation of restrictive care practices in these settings [37,38].

All retrieved articles will be imported into COVIDENCE software [40] for screening purpose and to remove duplications. Covidence is a user-friendly software designed to streamline the screening process for systematic reviews, offering features like bulk citation uploads, easy collaboration, and efficient data extraction [40]. Its strengths lie in its intuitive interface and time-saving automation, making it particularly useful for large-scale reviews. However, it has limitations, including restricted customization options for screening tools and occasional challenges with citation management. Two authors (ZB and BM) will independently screen the titles, abstracts, and full texts of indentified reviews based on predefined inclusion criteria. Before the actual screening process, a blind review of a sample of papers will be conducted to ensure a shared understanding is reached for the eligibility criteria. Any potential discrepancies between the two reviewers will be resolved through discussion or consultation with a third reviewer.

**Fig 2:**
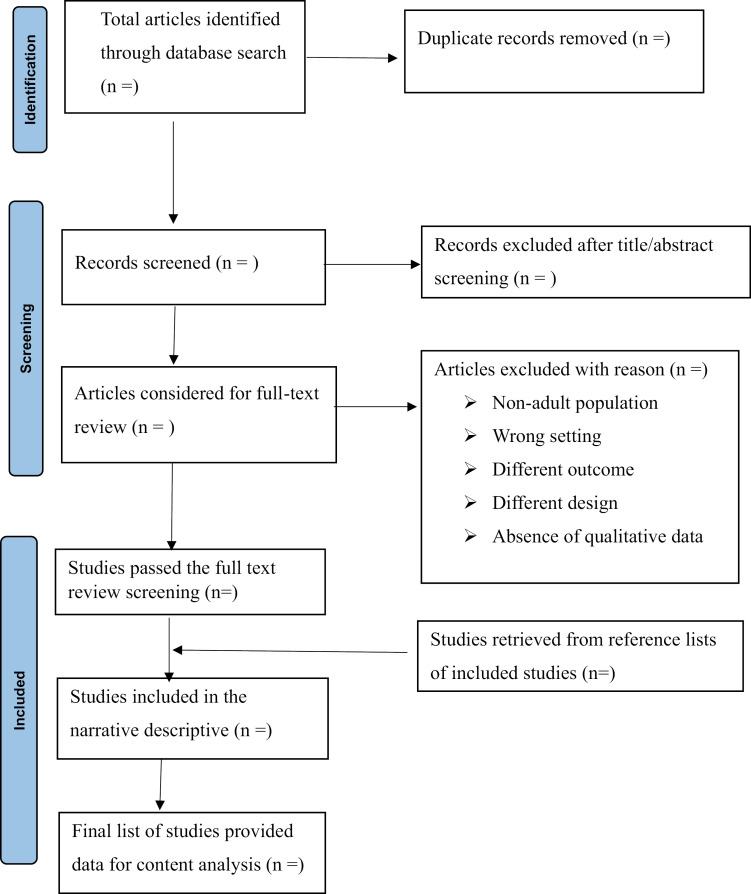
The PRISMA flow chart.

Finally, the research results and study selection processes, including the number of studies excluded at each screening stage and total nurmbers of studies included will be presented following the Preferred Reporting Items for Systematic Reviews and Meta-Analyses (PRISMA) 2020 guidelines [32] (**Fig 2**).

### Review Outcomes

The primary outcome of the planned umbrella review are any factors,  issues or contexts involving in healthcare professionals’ decision-making regarding the use of restrictive care practices. Restrictive care practices are broadly defined as interventions and measures that limit an individual’s freedom of movement, choice, or independence, typically employed to manage acute behavioral disturbances and ensure safety. This review will encompass multiple forms of restrictive care practices, including physical or mechanical restraint, seclusion, chemical restraint, involuntary admission, forced medication, and any other coercive measures. These practices are defined as follows:

- **Mechanical Restraint:** The use of mechanical devices (e.g., wrist or ankle cuffs, belts, chains, ropes, cloths or other devices) and/or attaching weighted items adjacent to the person’s body part to restrict an individual’s movement [1].
- **Physical/manual restraint:** The use of physical force or pressure, such as handholding or applying body weight, typically employed to prevent harm to an individual or others when they display behaviours that pose a risk of injury or danger. Physical/manual restraint may involve holding a person in a specific position, like a seated or standing hold, to temporarily control or restrict their movement.
- **Chemical Restraint:** The use of medication or drugs, not in the standard treatment (or dosage) to control or limit a person’s behaviour and limit the individual’s ability to function normally [1].
- **Seclusion:** Isolating an individual in a designated room or area by physically preventing them from interacting with other patients and staff to manage acute behavior where their ability to leave/exit the designated area or room by their own initiative has been restricted [1].
- **Involuntary Admission:** The process by which an individual is admitted to a mental health facility without their consent, typically due to concerns about their safety or the safety of others based on criteria related to mental health and risk [41].
- **Forced Medication:** Administering medication(s) to an individual without their consent, often used to manage severe symptoms or behavior that poses a risk to the individual or others in situations where the person is unable or unwilling to take medication voluntarily [1].

### Plan for quality assessment

A quality assessment will be conducted on all studies that meet the inclusion criteria using the JBI Critical Appraisal Checklist for Systematic Reviews [42]. This checklist comprises eleven questions that evaluate various methodological aspects, including clearly stated objectives, appropriateness of study inclusion criteria, adequacy of the literature search strategy, justification of study selection methods, assessment and documentation of study quality, and suitability of methods employed to synthesise findings. Each criterion is designed to ensure a rigorous and transparent execution of the systematic review. Responses to the checklist will be categorized as “Yes,” “No,” “Unclear,” or “Not Applicable,” with a score of one assigned for each “Yes” and zero for other responses (**Table 3**). A double appraisal will be independently conducted by two authors, and any discrepancies identified between the two assessors will be resolved through the involvement of a third author. Studies scoring less than 50% on the JBI Critical Appraisal Checklist will be excluded, thereby enhancing the robustness of the review and providing a more nuanced understanding of the factors influencing healthcare professionals’ decision-making in the context of restrictive care practices [43].

**Table 3:** The JBI critical appraisal checklist for systematic reviews.

Assessment questions	Scoring options			
	Yes	No	Unclear	Not Applicable
Is the review question clearly and explicitly stated?				
Were the inclusion criteria appropriate for the review question?				
Was the search strategy appropriate?				
Were the sources and resources used to search for studies adequate?				
Were the criteria for appraising studies appropriate?				
Was critical appraisal conducted by two or more reviewers independently?				
Were there methods to minimize errors in data extraction?				
Were the methods used to combine studies appropriate?				
Was the likelihood of publication bias assessed?				
Were recommendations for policy and/or practice supported by the reported data?				
Were the specific directives for new research appropriate?				

### Plan for data extraction

We planned the data extraction to be carried out using a Microsoft Excel spreadsheet, which will be initially prepared through collaboration among all authors. To ensure that the format effectively captures data addressing the research questions and accommodates various data presentations, the data collection template will be pilot tested with a subset of included studies. For the primary outcomes, textual information and discussions related to factors or issues influencing professionals’ decision-making regarding the use of restrictive practices in adult mental health inpatient settings will be extracted from each study and entered into the spreadsheet. In cases where such information is explicitly stated in the studies under review, we will also consider implicit statements to ensure a comprehensive understanding of implied messages and maintain data validity. Explicit statements are clear, direct, and straightforward expressions of a thought, idea, or piece of information, whereas implicit statements are messages or ideas that are suggested or hinted at rather than directly expressed, even though they are not directly stated [44]. The study is designed to gather data from research that employed a combination of qualitative, quantitative, or mixed methods, as long as they provided qualitative evidence related to factors influencing healthcare professionals’ decision-making in the implementation of restrictive care practices. For quantitative studies, we will adopt alternative approaches to extract qualitative data by focusing on the reported meanings rather than numerical values and converting these into qualitative components related to the factors influencing healthcare professionals’ decision-making.

Additionally, original characteristics of the reviews, including first author names, publication years, details on review methods (such as the type and number of databases searched, number of primary studies included, analysis methods, and quality assessment), and primary study aims will be extracted. ZB and BM will independently conduct the data extraction, wresolving any disagreements through the involvement of a third author (BA) and a joint review of conflicting studies.

### Plan for data management and evidence synthesis

A thematic synthesis will be performed to identify and summarize the factors influencing professionals’ decision-making. This approach is chosen to systematically integrate qualitative findings across multiple studies, allowing for a deeper understanding of the complex and often nuanced factors that guide professionals in their decision-making processes. Thematic synthesis is particularly well suited for this purpose because it enables the identification of common themes, patterns, and variations across different contexts, while also capturing the richness of the qualitative data. This method facilitates the generation of new insights and theoretical understandings that can inform practice, policy, and future research in mental health care.

The analysis will involve different steps to categorize and examine thematic elements of factors involved in the decision-making process [45,46]. Initially, verbatim text describing any issues influencing healthcare professionals’ decision-making regarding the use of restrictive care practices will be extracted from each study and presented in table formats. Researchers will read and re-read the extracted text to gain a comprehensive understanding of the overall message and implications of the extracted data. This process includes identifying and listing the relevant variables and their meaning units from the data. The identified variables will then be separately coded, which involves labeling or tagging data segments that represent specific concepts or themes. These codes will be condensed into broader themes that encompass multiple codes. The condensed themes will be further categorized into emergent themes by identifying commonalities and patterns among them, thus creating higher-order categories.

### Plan for result presentation

Once the umbrella review is done and results are generated, the findings for the proposed umbrella review will be presented in line with the Preferred Reporting Items for Overviews of Reviews (PRIOR) checklist, which is the preferred checklist to report umbrella reviews in health care [47] (S2 File). This approach can help improve the accuracy, completeness, and transparency of overviews [48]. This, in turn, could help maximize the value and impact of overviews by allowing more efficient interpretation and use of their research findings. The presentation will provide a concise overview of the major findings, highlighting key factors influencing professionals’ decision-making regarding restrictive care practices and their implications for clinical practice and policy.

The general characteristics of the included studies and their quality assessment scores will be reported using narrative descriptions and frequency tables. The synthesized qualitative evidence will be thoroughly presented, categorized into thematic areas, and summarized with textual narratives. Each theme will be explored in depth, supported by illustrative quotes and examples from the reviewed literature to provide context and nuance. The thematic elements identified in this review will be summarized in a figure or diagram to provide readers with clear, precise insights into the contexts that influence individual decision-making. This visual representation will enhance the clarity of the paper by highlighting key factors and their impact on decision-making processes. The findings will be interpreted in relation to previous studies, highlighting practical implications, policy recommendations, necessary practice changes, training needs, and suggesting promising areas for further research.

## Discussion

The proposed protocol aims to explore the factors that influence professionals’ decision-making in the use of restrictive care practices, such as restraint and seclusion, in adult mental health inpatient settings. There are diverse views and perspectives surrounding mental health in general, and restrictive care practices specifically [49]. These varying perspectives may lead professionals to manage mental health conditions differently, resulting in the varied application of restrictive interventions across mental health settings. Therefore, understanding these factors is essential for improving patient care, upholding ethical standards, and reducing the use of restrictive practices [50].

Restrictive practices are often employed in response to challenging or dangerous behaviors, yet their use raises significant ethical and clinical concerns [51]. Decisions to implement such practices are influenced by various factors, including organizational policies, staff training, cultural norms, legal frameworks, and individual patient characteristics [52]. By systematically examining these factors, the protocol seeks to identify the underlying reasons professionals resort to restrictive measures and to assess whether these decisions align with best practices and patient-centered care principles [53]. The study is designed to collect data through a combination of qualitative and quantitative methods, allowing for a comprehensive analysis of the decision-making process. This mixed-method approach will facilitate an in-depth understanding of both the explicit and implicit factors that guide professionals in their practice [54]. Additionally, it will explore the role of contextual issues, such as staff-to-patient ratios, resource availability, and organizational culture, in shaping decisions.

### Ethical dilemmas in professionals’ decision-making

There are diverse perspectives and attitudes toward restrictive care practices (RCPs). While some healthcare professionals view them as necessary interventions to manage behavior and ensure safety, others see them as intrusive measures infringing on individual rights [55]. This discord extends beyond healthcare professionals to service users and caregivers, who may hold differing opinions on RCPs. For example, some patients may prefer sedation over physical restraint, while caregivers might have conflicting views on what is best [94]. These complexities require professionals to navigate competing values, balancing ethical considerations, safety concerns, and the needs and preferences of service users when making decisions about RCPs [94].

Policies, protocols, and legal definitions guiding the implementation of restrictive care practices vary widely. As a result, healthcare professionals often rely on their subjective interpretations, which can lead to legal and ethical dilemmas when making decisions about restrictive care practices. These dilemmas arise from conflicting values, such as respecting patient autonomy versus ensuring the safety of others in the care environment. For instance, the decision to use chemical restraint might prioritize immediate safety but infringe upon a patient’s autonomy and dignity. Furthermore, cultural, organizational, and legal differences across regions complicate decision-making, leaving professionals uncertain about the most ethically sound and legally compliant actions. These challenges highlight the need for a nuanced understanding of how ethical principles and legal frameworks influence professionals’ decisions across various settings.

This study addresses these ethical and legal dilemmas by conducting internationally focused umbrella reviews to synthesize evidence from systematic reviews across diverse regions. By highlighting global patterns and contextual factors influencing decision-making in adult mental health inpatient settings, it will provide insights into how healthcare professionals approach RCPs. This evidence can inform the development of universally applicable yet regionally sensitive guidelines, shedding light on ethical considerations, cultural norms, and regulatory policies. By offering a global perspective, this approach aims to guide professionals toward informed, consistent, and equitable practices in restrictive care [56]. Policymakers may use the insights to revise guidelines, ensuring that restrictive practices are used as a last resort and within a framework of accountability and transparency, ultimately improving mental health care quality and protecting patients’ rights in inpatient settings [57].

### Strengths and limitations of the review

One of the key strengths of this review is its international focus, which includes literature from various countries and considers multiple types of restrictive care practices. This broad approach allows for a comprehensive understanding of the factors influencing professional decision-making, as some factors may apply only to specific forms of restrictive practices. Including various types of restrictive care practices enhances the depth of insights gained. Another strength is that the review adhered to PRISMA guidelines, conducted a quality appraisal of the studies, and registered its protocol with PROSPERO, ensuring transparency and rigor. This study has certain limitations. The main limitation of this review is the exclusion of non-English studies. This decision was made due to the absence of multilingual authors on the team and the lack of funding to hire professional interpreters. However, studies published in English or those with English-language versions will be included, without geographical restrictions, to provide a global perspective. Additionally, this review adopts a broader scope by including various forms of restrictive care practices such as physical restraint, seclusion, mechanical restraint, chemical restraint, involuntary admission, forced medication, and other coercive measures, offering a comprehensive understanding of the diverse contexts influencing healthcare professionals’ decisions. The study will not include restrictive care practices in other settings such as geriatric psychiatry, pediatrics, forensic units, emergency departments, and other educational environments, and this may limit the generalizability of the findings to non-adult populations. While the insights from this review will be useful for these contexts, we recommend that similar studies be conducted in these areas to enable comparisons with adult mental health inpatient settings. This would help foster a shared understanding and consistent terminology for the implementation and management of restrictive care practices across different sectors.

### Plan for dissemination of review findings

The findings of this review will be disseminated through presentations at various conferences, including the Annual Society for Mental Health Research (SMHR) Conference and the Mental Health Services (TheMHS) Conference. Additionally, the results will be submitted for publication in esteemed, peer-reviewed international journals, such as the International Journal of Mental Health Nursing. This approach is designed to maximize the impact and reach of the research, ensuring that the insights and recommendations are widely shared and accessible to a global audience.

## Supporting information

S1 File PRISMA for systematic review protocols (PRISMA-P) Checklist. (DOCX)

S2 File Preferred Reporting Items for Overviews of Reviews (PRIOR) Checklist. (PDF)
